# Effects of melon yellow spot orthotospovirus infection on the preference and developmental traits of melon thrips, *Thrips palmi*, in cucumber

**DOI:** 10.1371/journal.pone.0233722

**Published:** 2020-06-01

**Authors:** Shuhei Adachi-Fukunaga, Yasuhiro Tomitaka, Tamito Sakurai

**Affiliations:** 1 Kyushu Okinawa Agricultural Research Center, National Agriculture and Food Research Organization, Kumamoto, Japan; 2 Agricultural Research Center, National Agriculture and Food Research Organization, Ibaraki, Japan; Chinese Academy of Agricultural Sciences Institute of Plant Protection, CHINA

## Abstract

Melon yellow spot orthotospovirus (MYSV), a member of the genus *Orthotospovirus*, is an important virus in cucurbits. *Thrips palmi* is considered the most serious pest of cucurbits because it directly damages and indirectly transmits MYSV to the plant. The effects of MYSV-infected plants on the development time, fecundity, and preference of the thrips were analyzed in this study. Our results showed that the development time of male and female thrips did not differ significantly between MYSV-infected and non-infected cucumbers. The survival rate of thrips in non-infected and MYSV-infected cucumbers were not significantly different. In a non-choice assay, *T*. *palmi* adults were released on non-infected and MYSV-infected cucumbers and allowed to lay eggs. The number of hatched larvae did not significantly differ between non-infected and MYSV-infected cucumbers. In a choice assay, MYSV had no detectable effect on the number of adult thrips and preceding hatched larvae. In a pull assay, the settling rate of thrips on the released plant did not differ significantly when the adult thrips were released to non-infected or MYSV infected cucumbers for any cucumber cultivar. Based on our results, we propose that the effects of MYSV-infected cucumbers on the development time, fecundity, or preference of *T*. *palmi* may not be an important factor in MYSV spread between cucumbers.

## Introduction

Orthotospoviruses are species in the genus *Orthotospovirus* of the family *Tospoviridae*, and are transmitted by insect vectors, namely thrips, in a persistent, propagative manner [1, 2, 3]. Orthotospoviruses have a spherical enveloped virion, ca 80–110 nm in diameter, containing three single-strand RNA segments (L, M, and S) [4], and are divided into two major groups, Asia and Americas, based on the phylogeny of amino acid sequences of their nucleocapsid protein [5]. Melon yellow spot orthotospovirus (MYSV), a member of the genus *Orthotospovirus*, is an important virus in cucurbits and falls into the Asian group. MYSV was first reported in 1992 in Japan [6, 7]. MYSV mainly damages cucurbit crops such as watermelons (*Citrullus lanatus* Thunb.), wax gourds (*Benincasa hispida* Thunb.), netted melons (*Cucumis*. *melo* L.), and cucumbers (*Cu*. *sativus*) [8, 9, 10]. In cucumber, MYSV causes necrotic spots, yellowing, and mosaic symptoms, followed by loss of productivity and quality [6, 9, 10, 11]. Moreover, it is difficult to control MYSV because it infects not only cucurbits but also weeds [12].

Thrips are widely distributed throughout the world and have evolved into more than 6000 species. They are categorized based on different feeding types, such as flower feeders, mature-leaf feeders, and young-leaf feeders [13, 14]. *Thrips palmi* Karny (Thysanoptera: Thripidae) is a leaf feeder and is one of the most important species to transmit orthotospoviruses, such as calla lily chlorotic spot orthotospovirus (CCSV), groundnut bud necrosis tospovirus (GBNV), melon yellow spot orthotospovirus (MYSV), and watermelon silver mottle tospovirus (WSMoV) [13, 15]. *T*. *palmi* was first reported in 1925 in Indonesia and has been recognized as a pest of various agricultural and horticultural plants since the late 1970s, followed by worldwide invasion [16, 17]. In Japan, *T*. *palmi* was first confirmed in the Kyushu region in 1978 and is one of the most important pests of cucumber (*Cu*. *sativus* L.), eggplant (*Solanum melongena* L.), and green pepper (*Ca*. *annuum*) in the western part of Japan [18]. In Japan, *T*. *palmi* causes serious losses in cucumber yield. For example, the tolerable *T*. *palmi* density for cucumber was estimated at 4.4 adult per leaf for uninjured fruit yield, assuming a permissible yield loss level of 5% [19]. Recently, the emergence of insecticide-resistant *T*. *palmi* has made it difficult to control them in Japan [20].

Most plant viruses are transmitted by insects, including thrips [21]. The viruses induce chemical and physical changes in the host plant, allowing efficient transmission from plant to plant [22, 23, 24, 25, 26, 27]. Numerous studies on the interaction between viruses and vector insects have been reported so far [28, 29]. The interactions between orthotospovirus and thrips have been widely analyzed in tomato spotted wilt tospovirus (TSWV) and its vector, *Frankliniella occidentalis* (Pergande) [30, 31]. Several studies have described the positive effects of TSWV on *F*. *occidentalis* larvae via the host plant, such as a shorter development time and higher survival rate [32, 33, 34, 35]. In contrast, complicated effects of TSWV have been reported in adult *F*. *occidentalis*. We previously showed that jasmonate (JA) plays an important role in a plant’s response and resistance to *F*. *occidentalis*, and that the JA-regulated plant defense negatively affects the performance of *F*. *occidentalis* and their preference [36, 37]. We also reported that TSWV-infected Arabidopsis plants attracted *F*. *occidentalis* and indicated the importance of balance among plant defense systems for its attraction [22, 38].

Unlike the TSWV (Americas group) and *F*. *occidentalis* (*Frankliniella* genus) interaction, few studies have focused on the interaction between the orthotospovirus (Asia group) and *T*. *palmi* [39, 40]. Overall, the interactions between other orthotospoviruses and *T*. *palmi* remain largely unknown. MYSV belongs to the Asia group and is distantly related to TSWV of the Americas group. *T*. *palmi* is a leaf feeder classified into the genus *Thrips* and is distantly related to *F*. *occidentalis*. Cucumber is known as the most suitable host plant for both, MYSV and *T*. *palmi*, among other cucurbit species because the plant supports a high development rate and survival of *T*. *palmi* [41, 42, 43]. Therefore, physiological and nutritional traits of the MYSV-infected cucumber plants may affect the developmental traits and feeding preference of *T*. *palmi*. The analysis of the interaction between MYSV and *T*. *palmi* is useful for a deeper understanding of interactions between orthotospoviruses and thrips. Moreover, studies on the interaction between MYSV, *T*. *palmi*, and cucumber will shed light on how the MYSV is spread by thrips in the field. Therefore, we examined the indirect effects of MYSV on the developmental traits and preference of *T*. *palmi* in cucumber plants.

## Materials and methods

### Insects

Two laboratory reared strains of non-viruliferous *T*. *palmi* were obtained from a cucumber plant in Koshi city, Kumamoto Prefecture in 2018 (Kumamoto strain) and an egg plant in Nankoku city, Kochi Prefecture in 2002 (Kochi strain). These strains were maintained on cucumber cv. Natsusuzumi (Takii Seed Co., Ltd., Kyoto, Japan) in insect chambers at 25°C and 16-h light:8-h dark photoperiodic conditions. Cucumbers at the two-true leaf stage were enclosed with thrips in a plastic cage (25 cm length × 30 cm width × 28 cm height). The plants were replaced with new seedlings every 2–3 weeks. The non-viruliferous *T*. *palmi* colonies were used for the following experiments.

### Viruses and plants

The two MYSV isolates MYSV-FuCu05P [44] and MYSV-E08k were isolated from diseased cucumbers in Fukuoka and Ehime Prefectures, respectively. The virus was mechanically inoculated onto the fully expanded cotyledons of cucumbers cvs. Natsusuzumi, Excellent fushinari-2go (Saitama Gensyu Ikuseikai Co., Ltd., Saitama, Japan) or Tokiwa (Tokiwa Co., Ltd., Saitama, Japan). After 14 days, systemically infected leaves were harvested and stored at -80°C until use.

For mechanical inoculation of the virus, MYSV-infected leaves were ground in a mortar and pestle with 0.05 M phosphate buffer (pH 7.0) containing 0.01 M sodium sulfite. The sap was inoculated onto the cotyledons of cucumbers in the 1-true leaf developmental stage. Healthy plants treated with the buffer were used as a control. The plants were grown a greenhouse at 28°C under natural light conditions. Approximately 13 days later, necrotic spots appeared on the cotyledons, and these plants were used for subsequent experiments as MYSV-infected plants. In addition, MYSV-infection was confirmed by double-antibody sandwich enzyme-linked immunosorbent assay (DAS-ELISA) using polyclonal antibodies raised against the N protein of MYSV (Japan Plant Protection Association) in choice and pull assays after the experiments. DAS-ELISA was conducted according to the manufacturer’s instructions. A leaf sample with an optical density greater than three times the mean of the non-infected controls was considered positive.

### Development time and survival rate

2–3 non-infected cucumbers cv. Natsusuzumi were separately placed in plastic cages (24 cm length × 24 cm width × 32 cm height). Ten to twenty adult males and the same number of adult female thrips (Kumamoto strain) were released on the plants and allowed to lay eggs. All adults were removed from the plants after 24 h and the hatched larvae (1-day-old) were used for the experiments.

For the development time and survival rate assay, leaves sections (10 mm length × 10 mm width) were prepared from second or third expanded true leaves of cucumber cv. Natsusuzumi with scissors. MYSV-infected (MYSV-FuCu05P) and non-infected leaves were floated (adaxial side up) on 1 mL of distilled water in each well of a 24-well tissue culture plate (Sumitomo Bakelite Co. Ltd) to restrict thrips feeding to the upper surface of the leaves, as described in a previous study [45]. Hatched larva (1-day-old) was placed onto each leaf and the wells were covered with MicroAmp Optical Adhesive Film (Applied Biosystems, Foster City, CA, USA). These 24-well tissue culture plates were put in an insect chamber at 25°C under 16-h light:8-h dark photoperiodic conditions. The developmental stage of the thrips was checked by visual observation every 24h with a stereoscopic microscope (SMZ745T, NIKON Corporation, Tokyo, Japan) until adult eclosion. Each *T*. *palmi* was transferred onto a new leaf with a small brush as previously described [40] every 72h and the survival of the individuals was also checked at the same time. The experiments were conducted three times with 18–24 leaves, then 66 replicates were prepared in MYSV and non-infected treatments, respectively. When the individuals accidentally dropped into the water or disappeared from the well, they were removed from the data. A total of 56 and 57 leaves were used for MYSV-infected and non-infected treatments, respectively, to measure the survival rate. Any dead individual at any developmental stage was not included when measuring the development time at a specific stage. Finally, for MYSV treatment, 23 and 29 leaves were used for measuring the development time of males and females, respectively, while for non-infected treatment, 18 and 32 leaves were used for measuring the development time of males and females, respectively.

Statistical differences in the effects of MYSV infection on the development time of *T*. *palmi* were analyzed by Wilcoxon rank sum test because the data were not normally distributed at all stages and sexes (Shapiro-Wilk normality test, p < 0.001). The effects of MYSV infection on the survival rate of *T*. *palmi* were analyzed by Fisher’s exact test. These analyses were conducted using R version 3.1.0. (The R foundation for statistical computing, Vienna, Austria).

### Number of hatched larvae in the non-choice assay

Oviposition preference of *T*. *palmi* adults was examined using MYSV-infected and non-infected cucumbers. A MYSV-inoculated (MYSV-FuCu05P isolate) and a non-inoculated plant, *Cu*. *sativus* cv. Natsusuzumi were separately placed in plastic cages (24 cm length × 24 cm width × 32 cm height). Five male and female adult *T*. *palmi* (Kumamoto strain) were released on each plant and allowed to lay eggs. After three days, all adult thrips were removed from the plants. The number of hatched larvae on each plant was counted to assess the oviposition preference of *T*. *palmi*. The experiment included 11 and 12 replicates on MYSV-infected and non-infected treatments. All experiments were conducted in an insect chamber at 25°C under 16-h light:8-h dark photoperiodic conditions. Differences in the number of hatched larvae in MYSV-infected and non-infected cucumber plants were analyzed by Student–s *t*-test with R version 3.1.0.

### Choice assay

The comprehensive preference of *T*. *palmi* adults for MYSV-infected cucumbers was measured in a choice assay. Cucumber cv. Kuraju was used for this assay. A MYSV-inoculated (MYSV-E08k isolate) and non-inoculated cucumbers were placed in a plastic cage (60 cm length × 45 cm width × 50 cm height) separated by 40 cm. Twenty adult females of *T*. *palmi* (Kochi strain) were released halfway between the plants. The number of adult thrips on each plant were then counted 1, 3, and 7 days later. In addition, the number of hatched larvae on each plant was counted after 7 days. The experiment was conducted twice with 4 plants in each treatment for a total of eight replicates in an insect chamber at 25°C under 16-h light:8-h dark photoperiodic conditions. The effect of time (days) and plant treatment on the number of adults was analyzed by two-way ANOVA. Statistical differences in the number of hatched larvae on the MYSV-infected and non-infected plant was analyzed by Student’s *t*-test. The statistical analyses were performed using R version 3.1.0.

### Pull assay

To further investigate the effect of MYSV infection on the behavior of *T*. *palmi* adults, a pull assay was conducted as described in a previous study [31]. A MYSV-E08k-inoculated and non-inoculated plant were placed in a plastic cage (45 cm length × 90 cm width × 45 cm height) separated by 40 cm. Ten adult male and 40 female thrips (Kochi strain) were released onto MYSV-infected or non-infected plants. After three days, the number of adult thrips on each plant was counted. This experiment was conducted once using three different cucumber cultivars, Tokiwa, Natsusuzumi, and Shakitto (Takii), under the following conditions: 25°C under 16-h light:8-h dark photoperiodic conditions. Statistical differences in the number of thrips on MYSV-infected and non-infected plants were analyzed by Fisher’s exact test with R version 3.1.0.

## Results

### Development time and survival rate

**Fig 1:**
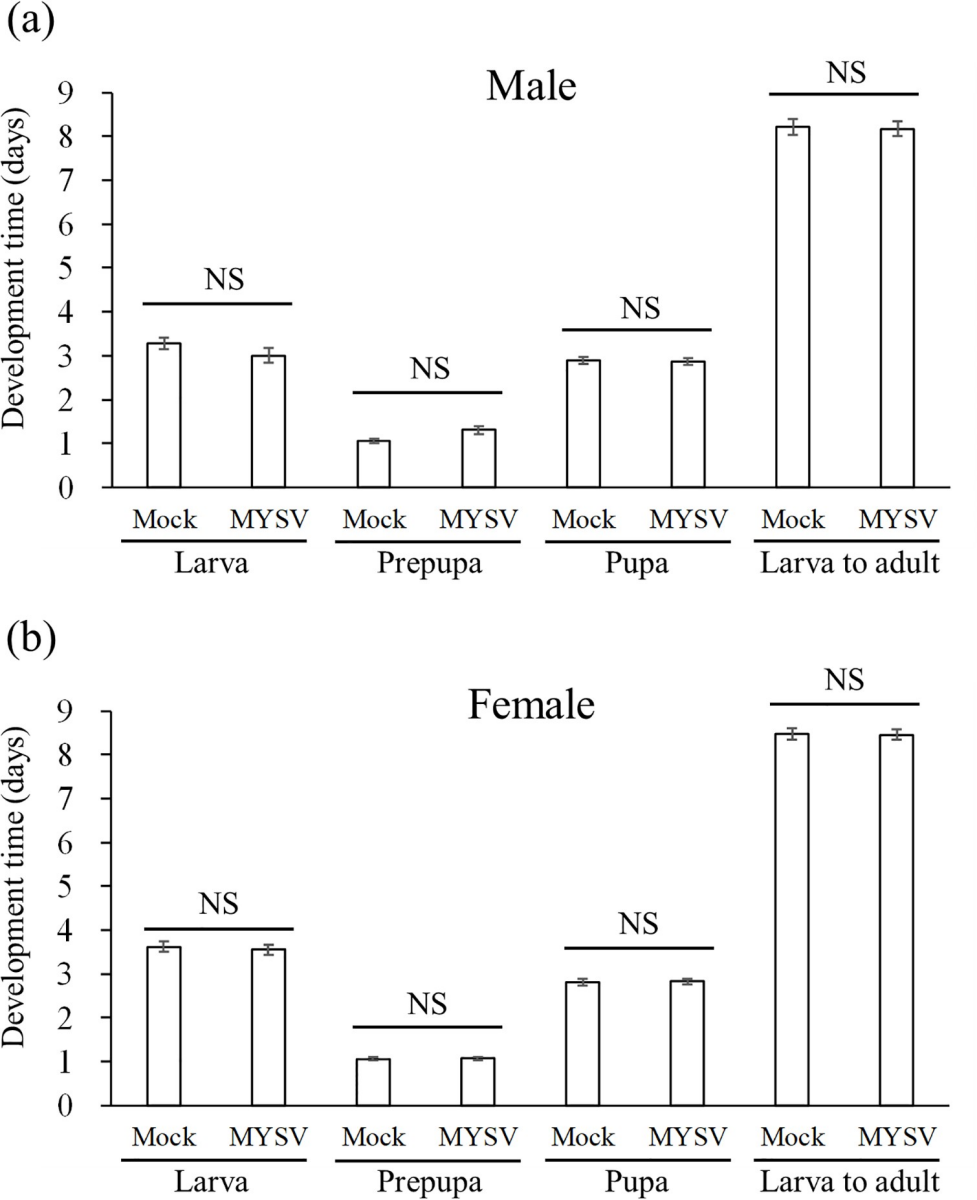
Development time of (a) male and (b) female *Thrips palmi* on non-infected (Mock) and MYSV-infected (MYSV) cucumbers in each developmental stage. A hatched larva was released onto each leaf and the total number of days for each stage was calculated. Development time for the first and second instar larval stages were summed together as the larva stage. Values are the means ± SE. “NS” indicates no significant difference between the treatments on a developmental stage (*p* > 0.05, Wilcoxon rank sum test).

We compared the development time and survival rate of *T*. *palmi* on MYSV-infected and non-infected plants. On non-infected plants, the times for each developmental stage of male thrips were 3.3 ± 0.1 days (larva), 1.1 ± 0.1 days (prepupa), 2.9 ± 0.1 days (pupa), and 8.2 ± 0.2 days (larva to adult). On MYSV-infected plants, the times were 3.0 ± 0.2 days (larva), 1.3 ± 0.1 days (prepupa), 2.9 ± 0.1 days (pupa), and 8.2 ± 0.2 days (larva to adult). There were no significant differences between non-infected and MYSV-infected plants; larva (*W* = 247, *p* = 0.26), prepupa (*W* = 156, *p* = 0.051), pupa (*W* = 211, *p* = 0.87), and larva to adult (*W* = 137, *p* = 0.45) (Fig 1a). On non-infected plants, the times for each developmental stage of female thrips were 3.6 ± 0.1 days (larva), 1.1 ± 0.04 days (prepupa), 2.8 ± 0.1 days (pupa), and 8.5 ± 0.1 days (larva to adult). On MYSV-infected plants, the times were 3.6 ± 0.1 days (larva), 1.1 ± 0.05 days (prepupa), 2.8 ± 0.1 days (pupa), and 8.4 ± 0.1 days (larva to adult). There were no significant differences between non-infected and MYSV-infected plants; larva (*W* = 496, *p* = 0.61), prepupa (*W* = 461, *p* = 0.93), pupa (*W* = 455, *p* = 0.85), and larva to adult (*W* = 140, *p* = 0.89) (Fig 1b).

**Fig 2:**
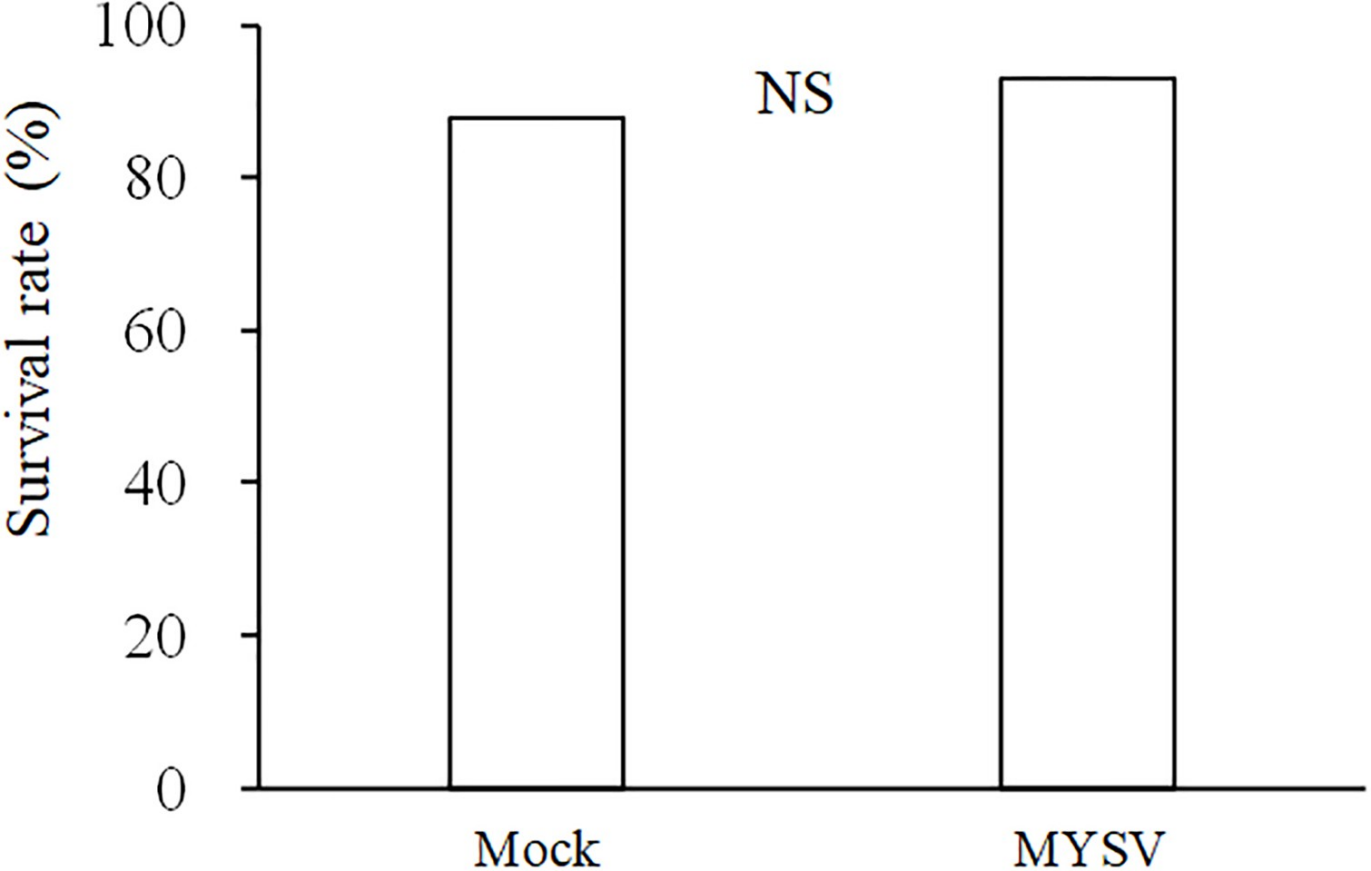
Survival rate of *Thrips palmi* from larva to adult on non-infected (Mock) and MYSV-infected (MYSV) cucumbers. “NS” indicates no significant difference between the treatments (*p* > 0.05, Fisher’s exact test).

The survival rates of thrips on non-infected and MYSV-infected cucumbers were 87.7% and 92.9%, respectively. There was no significant difference in the survival rate of thrips on non-infected and MYSV-infected plants (*p* = 0.52, Fisher’s exact test) (Fig 2).

### Number of hatched larvae in the non-choice assay

**Fig 3:**
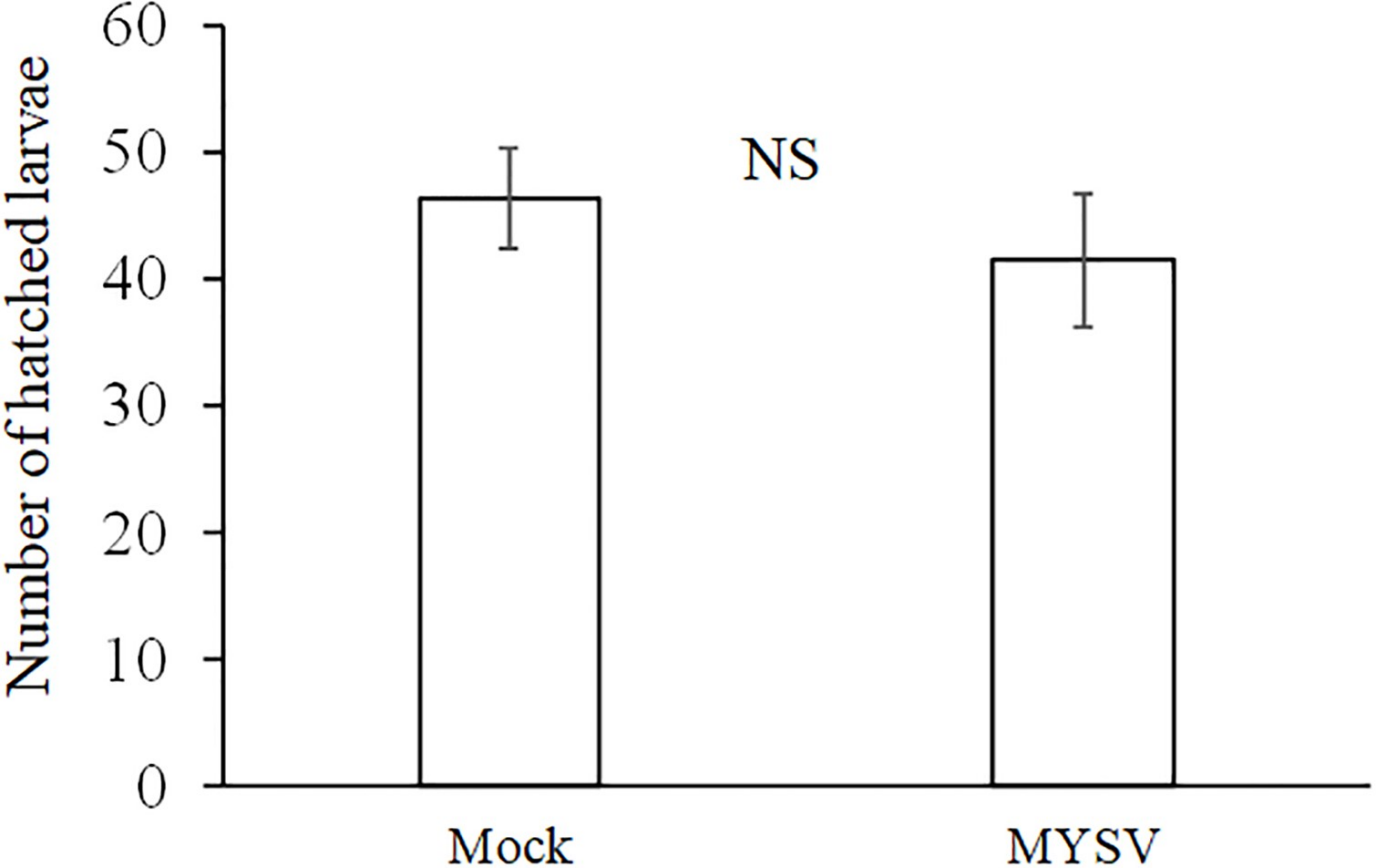
Number of hatched larvae in a non-choice assay between non-infected (Mock) and MYSV-infected (MYSV) cucumbers. Five adult male and female of *T*. *palmi* were released on non-infected or MYSV-infected cucumbers for 3 days. The total number of larvae were counted 6 days later. Values are the means ± SE. “NS” indicates no significant difference between the treatments (*p* > 0.05, Student–s *t*-test).

The effect of MYSV on oviposition of *T*. *palmi* was analyzed using MYSV-infected and non-infected plants. There were 46.2 ± 4.0 and 41.4 ± 5.2 hatched larvae on non-infected and MYSV-infected plants, respectively, showing no significant difference in the number of ovipositions on non-infected and MYSV-infected plants (Fig 3) (*t* = 0.73, *p* = 0.47).

### Choice assay

**Fig 4:**
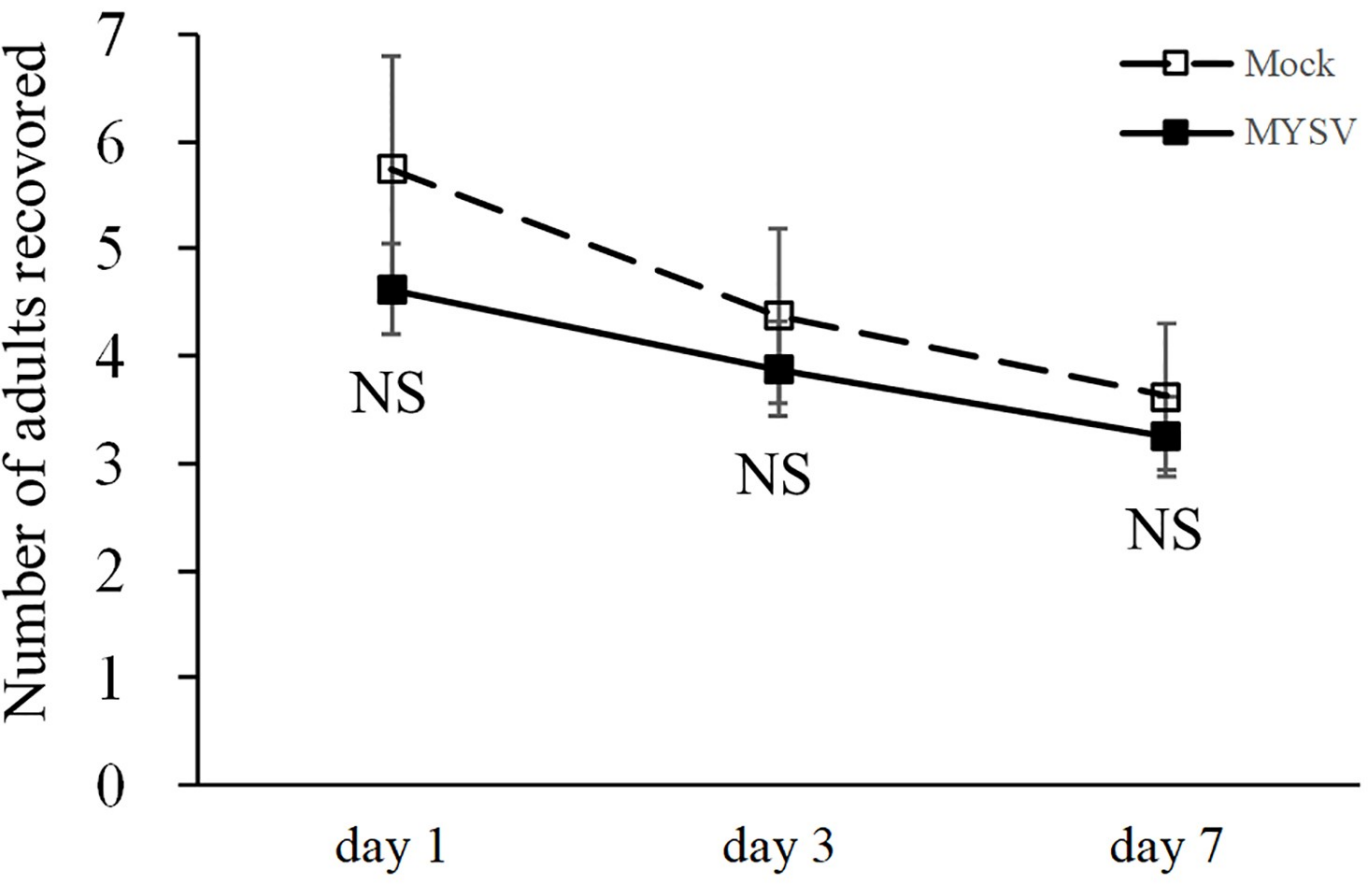
Number of *Thrips palmi* adults recovered in a choice assay between non-infected (Mock) and MYSV-infected (MYSV) cucumbers. Twenty adult thrips were released between non-infected and MYSV-infected cucumbers. The total number of adults on each cucumber was counted after days 1, 3, and 7 from release of the adult thrips. Both cucumbers were 40 cm apart from each other. Values are the means ± SE.

To investigate the preference of *T*. *palmi* for MYSV-infected plants, a choice assay was conducted. There were 5.8 ± 1.0, 4.4 ± 0.8 and 3.6 ± 0.7 adult thrips on non-infected plants at 1, 3, and 7 days after release, respectively. On MYSV-infected plants, there were 4.6 ± 0.4, 3.9 ± 0.4, and 3.3 ± 0.4 adult thrips at 1, 3, and 7 days after release, respectively (Fig 4). There was significant effect of time on the number of adult thrips, but plant treatment and the interaction effect on the number of adult thrips were not significant (Time: *F* = 3.4, df = 2, *p* < 0.05; Treatment: *F* = 1.4, df = 1, *p* = 0.23; Time × Treatment: *F* = 0.17, df = 2, *p* = 0.83) (Fig 4).

**Fig 5:**
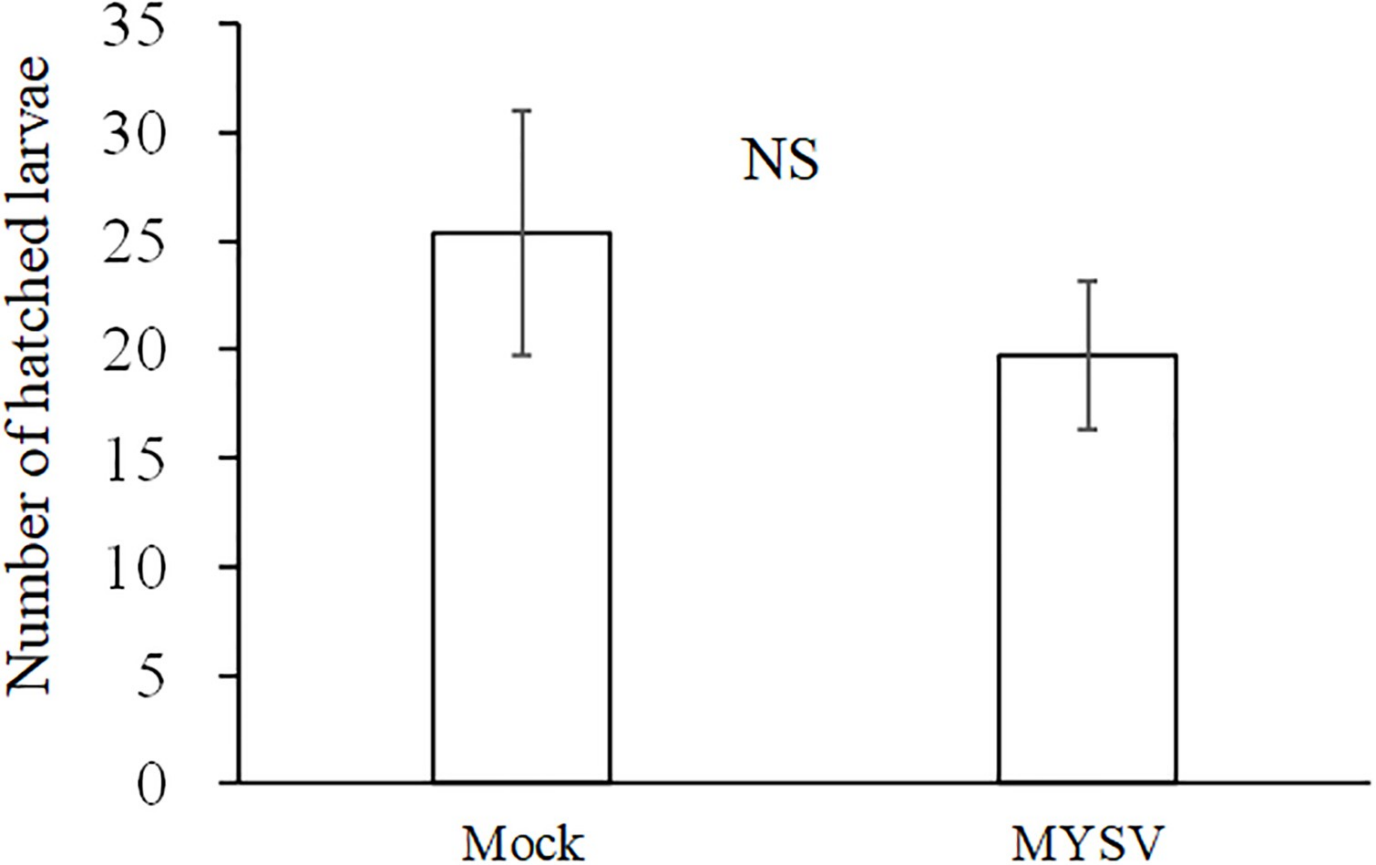
Number of hatched larvae in a choice assay between non-infected (Mock) and MYSV-infected (MYSV) cucumbers. Twenty *T*. *palmi* adults were released between non-infected and MYSV-infected cucumbers and allowed to lay eggs. The total number of hatched larvae on each cucumber was counted 7 days later. Both cucumbers were 40 cm apart from each other. Values are the means ± SE. “NS” indicates no significant difference between the treatments (*p* > 0.05, Student’s *t*-test).

There was no significant difference in the number of hatched larvae on non-infected and MYSV-infected plants at 7 days (*t* = 0.85, *p* = 0.41), with 25.4 ± 5.6 and 19.8 ± 3.4 hatched larvae on non-infected and MYSV-infected cucumbers, respectively (Fig 5).

### Pull assay

**Fig 6:**
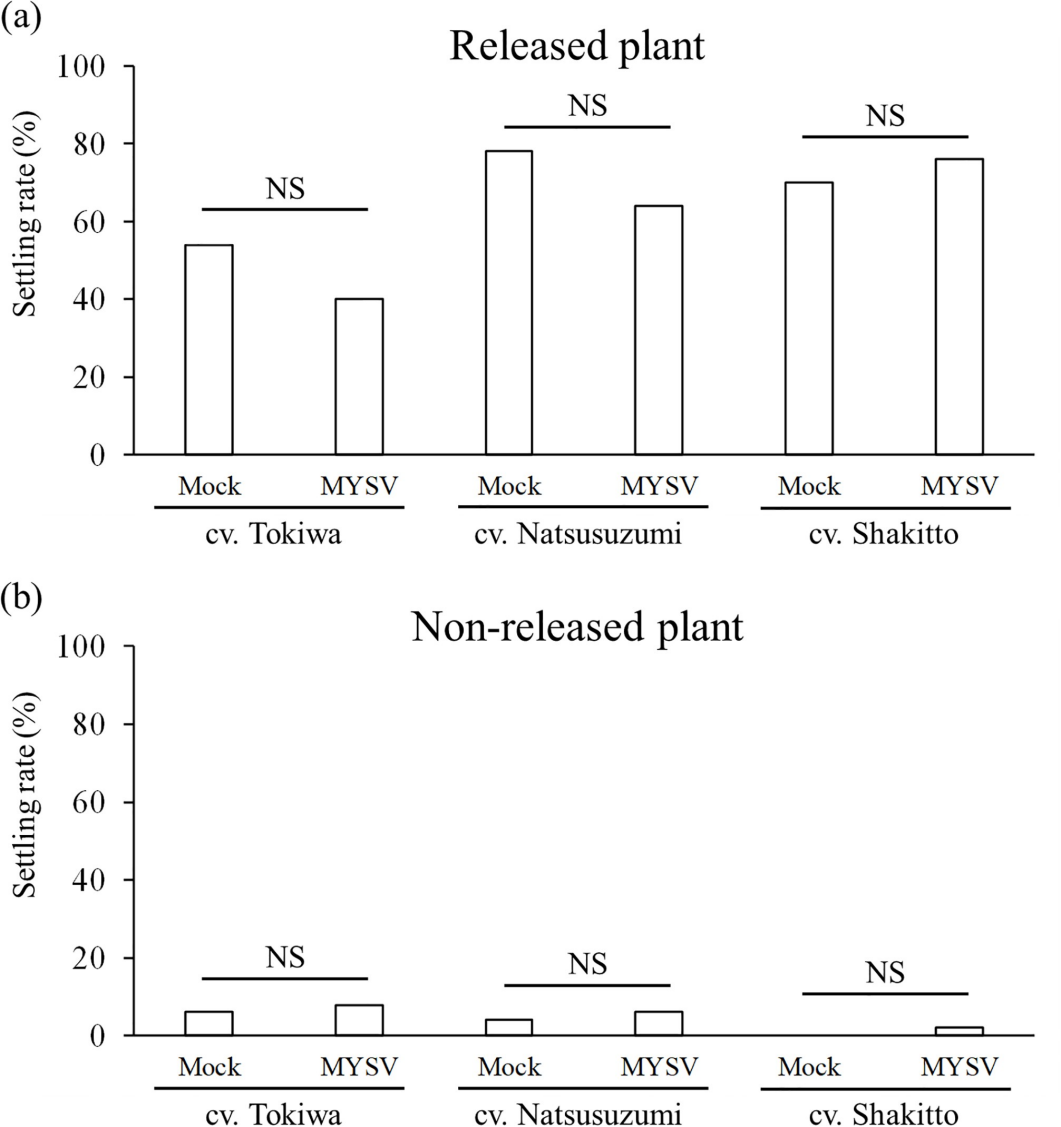
Settling rate of *Thrips palmi* adults when adults were released onto non-infected (Mock) or MYSV-infected (MYSV) cucumbers in a pull assay on each variety. The total number of adults on (a) released and (b) non-released cucumbers were counted 3 days from release of the adults. The cucumbers were 40 cm apart from each other. “NS” indicates no significant difference between the treatments on each variety of cucumber (*p* > 0.05, Fisher’s exact test).

A pull assay using three different cucumber cultivars was conducted to further investigate the effect of MYSV infection on the behavior of thrips on cucumbers. When adult thrips were released onto non-infected plants, the settling rates of the thrips on released plant were 54% (Tokiwa), 78% (Natsusuzumi), and 70% (Shakitto). When they were released onto the MYSV-infected plants, the rates were 40% (Tokiwa), 64% (Natsusuzumi), and 76% (Shakitto) (Fig 6a). There were no significant differences in the settling rate of thrips on non-infected and MYSV-infected plants; Tokiwa (*p* = 0.23), Natsusuzumi (*p* = 0.19), and Shakitto (*p* = 0.65) (Fig 6a).

When adult thrips were released onto non-infected plants, the settling rates of the thrips on non-released plant were 6% (Tokiwa), 4% (Natsusuzumi), and 0% (Shakitto). When they were released onto MYSV-infected cucumbers, the rates were 8% (Tokiwa), 6% (Natsusuzumi), and 2% (Shakitto) (Fig 6b). There were no significant differences in the settling rate of thrips on non-infected and MYSV-infected plants; Tokiwa (*p* = 1), Natsusuzumi (*p* = 1), and Shakitto (*p* = 1) (Fig 6b).

## Discussion

In the current study, we mainly examined the indirect effects of MYSV on *T*. *palmi*. Our results indicated that MYSV did not indirectly affect the development, oviposition, mobility, and preference of *T*. *palmi* in cucumber.

Previous studies on TSWV and *F*. *occidentalis* have suggested that the quality of pepper and Arabidopsis plants decreased after feeding by thrips, and this decreased the survival and development of *F*. *occidentalis* [32, 36, 37]. Crosstalk between the salicylic acid (SA) and JA pathways plays an important role in plant defense against herbivores [46, 47]. We previously revealed that *F*. *occidentalis* feeding induced JA in *Arabidopsis thaliana*, decreasing thrips performance such as feeding and oviposition. In contrast, TSWV infection induced SA, a plant hormone known to be antagonistic to JA, effectively decreasing the JA response and increasing thrips fitness [22, 36, 38]. However, these results differ from the results obtained in this study. The difference in feeding types between the thrips species may explain why MYSV did not affect the development of *T*. *palmi* in this study. Most TSWV and *F*. *occidentalis* studies have been conducted on plant seedlings without flowers [32, 36, 37]. As *F*. *occidentalis* is a flower feeder, TSWV infection may affect the feeding and development of *F*. *occidentalis* through the decrease in the defense response of the host plant. In other words, TSWV infection may play an essential role in the adaptation of *F*. *occidentalis* to the leaves. On the other hand, *T*. *palmi* did not require MYSV infection in the host plants. Thus, *T*. *palmi* may basically adapt to the plants, MYSV-infected or not, because *T*. *palmi* is originally a leaf feeder. In other words, the host plants may not induce deleterious metabolism in *T*. *palmi*, and, therefore, the development of *T*. *palmi* may not change between MYSV-infected and healthy plants. There is only one report stating that groundnut bud necrosis tospovirus (GBNV) infection positively affects the development of *T*. *palmi* in bean [40]. However, Kawai [41] showed that beans are of low quality for *T*. *palmi*, suggesting that orthotospovirus infection in low-quality plants for thrips is mainly beneficial to thrips performance, such as survival rate and fecundity. Virus-infected plants are often more attractive to vector insects than non-infected plants. Although the attractiveness to thrips varies depending on the combination of plant viruses and insects, the developmental traits of the thrips in the ‘attractive’ plants were positively affected in most cases. In fact, previous studies indicated that *F*. *occidentalis* prefers the cultivated tomato to wild types with higher levels of acylsugars, which are feeding and oviposition deterrents [48, 49]. Moreover, on the thrips-resistant cultivars of cucumber, *F*. *occidentalis* spends a lot of time walking to widely dispersed feeding sites when compared to susceptible cultivars, on which suitable feeding sites are more densely clumped [49]. Hence, thrips may prefer plants that are high-quality as a food source to develop the population. In other words, *T*. *palmi* may not prefer MYSV-infected cucumber because the host qualities are similar between non-infected and MYSV-infected cucumbers. The effects of orthotospovirus on the development and preference of thrips are more complicated among plants, viruses, and vector species. In order to understand the interaction between them, more detailed studies are needed to better understand the life history of orthotospoviruses.

The acquisition of orthotospovirus occurs in vector thrips at the larval stage [30]. Because some thrips larvae fail in virus acquisition [39], virus-infected thrips (directly and indirectly affected by orthotospovirus) and non-infected thrips (indirectly affected by orthotospovirus) are mixed on orthotospovirus-infected plants. Although we did not distinguish between the direct and indirect effects of MYSV on the developmental traits of *T*. *palmi*, our results indicated that MYSV infection did not affect the survival and development of *T*. *palmi* in cucumber. On the other hand, Chen et al. [39] examined the direct and indirect interaction of WSMoV and *T*. *palmi* using watermelon. The results showed that there were no significant differences in longevity and fecundity between WSMoV-infected and non-infected thrips. Thus, our results were consistent with those reported by Chen et al. [39].

Here, we showed a novel interaction between MYSV and *T*. *palmi*. In this study, our results showing that MYSV infection did not affect thrips behavior differ from the results of previous studies. The results raised the question of how efficiently did MYSV spread in the field. We have two hypotheses in response to this question. The most plausible suggestion is that MYSV can be efficiently transmitted by *T*. *palmi* without a change in vector preference because the thrips are highly adapted in cucumbers. Another hypothesis is that the spread of MYSV may be related to the quality of the leaves, which indicates the symptoms caused by MYSV infection. As the yellow and necrotic spot symptoms caused by MYSV infection develop rapidly in cucumber, *T*. *palmi* may rapidly migrate from the infected plants to healthy plants. Accordingly, MYSV is also able to rapidly migrate in the field. In any case, our results suggest that different mechanisms are involved in the interaction between MYSV, *T*. *palmi*, and cucumber. Further analyses are needed to understand the detailed mechanism of this tritrophic interaction. To the best of our knowledge, this is the first report of a novel interaction between an orthotospovirus and thrips, obtained by observing MYSV, *T*. *palmi*, and cucumber. This study will be useful for the development of novel means to control both *T*. *palmi* and MYSV.
